# A longitudinal study of higher-order thinking skills: working memory and fluid reasoning in childhood enhance complex problem solving in adolescence

**DOI:** 10.3389/fpsyg.2015.01060

**Published:** 2015-07-27

**Authors:** Samuel Greiff, Sascha Wüstenberg, Thomas Goetz, Mari-Pauliina Vainikainen, Jarkko Hautamäki, Marc H. Bornstein

**Affiliations:** ^1^Education, Culture, Cognition and Society Unit, University of LuxembourgLuxembourg, Luxembourg; ^2^University of KonstanzKonstanz, Germany; ^3^Thurgau University of Teacher Education, KreuzlingenSwitzerland; ^4^University of HelsinkiHelsinki, Finland; ^5^Eunice Kennedy Shriver National Institute of Child Health and Human Development, National Institutes of Health, Bethesda, MDUSA

**Keywords:** cognitive development, complex problem solving, problem solving, working memory, fluid reasoning

## Abstract

Scientists have studied the development of the human mind for decades and have accumulated an impressive number of empirical studies that have provided ample support for the notion that early cognitive performance during infancy and childhood is an important predictor of later cognitive performance during adulthood. As children move from childhood into adolescence, their mental development increasingly involves higher-order cognitive skills that are crucial for successful planning, decision-making, and problem solving skills. However, few studies have employed higher-order thinking skills such as complex problem solving (CPS) as developmental outcomes in adolescents. To fill this gap, we tested a longitudinal developmental model in a sample of 2,021 Finnish sixth grade students (*M =* 12.41 years, SD = 0.52; 1,041 female, 978 male, 2 missing sex). We assessed working memory (WM) and fluid reasoning (FR) at age 12 as predictors of two CPS dimensions: knowledge acquisition and knowledge application. We further assessed students’ CPS performance 3 years later as a developmental outcome (*N* = 1696; *M =* 15.22 years, SD = 0.43; 867 female, 829 male). Missing data partly occurred due to dropout and technical problems during the first days of testing and varied across indicators and time with a mean of 27.2%. Results revealed that FR was a strong predictor of both CPS dimensions, whereas WM exhibited only a small influence on one of the two CPS dimensions. These results provide strong support for the view that CPS involves FR and, to a lesser extent, WM in childhood and from there evolves into an increasingly complex structure of higher-order cognitive skills in adolescence.

## Introduction

“Study the past if you would define the future.”

Confucius (551 – 479 BC)

This quote from the Chinese philosopher Confucius offers an astonishingly accurate reflection of the central message found in current research on the development of human cognition: early performance levels on cognitive ability tests strongly predict later test performance, thus recognizing that cognitive skills in infancy provide a window into cognitive ability later (Bornstein et al., 2006, 2013; Rose et al., 2008). For instance, early indicators of information processing at the age of 12 months predict levels of intellectual functioning at the age of 48 months on verbal and non-verbal tests of cognition (Blaga et al., 2009). Indeed, a wealth of developmental studies has contributed to the extant body of literature that highlights the stability of cognitive performance from infancy to adolescence and even beyond (Kavsek, 2004; Bornstein et al., 2006, 2013; Demetriou et al., 2008). Thus, there is conceptual and empirical agreement that early cognitive performance is highly relevant for intellectual functioning and cognitive performance later in life (Bornstein, 2014). However, it is also acknowledged that there is room for change and that this relation is not deterministic (e.g., Hoff and Tian, 2005; Tong et al., 2007; Bornstein et al., 2013).

The majority of the extant empirical research on the development of human intellect views abilities, such as fluid reasoning (FR) or working memory (WM), as the final developmental outcomes (see Bornstein, 2014 for an overview). However, when children enter adolescence, it is well acknowledged that cognitive development increasingly involves higher-order cognitive processes that transcend FR, such as planning, inhibitory control, decision-making, and problem solving (Asato et al., 2006; Conklin et al., 2007). For instance, Asato et al. (2006) found that inhibitory control was the strongest predictor of performance on the problem solving task Tower of London in participants between the ages of 8 and 30 and that inhibitory control demonstrated strong maturation during adolescence (cf. Albert and Steinberg, 2011). That is, as the human mind unfolds, it advances from applying fundamental information-processing skills all the way to using complex higher-order thinking processes, and this development continues well into adolescence (Galotti, 2011). Consequently, higher-order thinking processes are considered crucial determinants of life success and lifelong learning (Autor et al., 2003; OECD, 2012). For instance, Mayer and Wittrock (2006) highlight that making students good problem solvers is one of the greatest challenges in education, and they stress knowledge of how problem-solving skills evolve as one of the central questions currently faced by scientists around the globe. Important for this study, only a few empirical efforts have integrated higher-order thinking skills as outcome variables into developmental studies of the human mind. That is, although our understanding of the development of cognition in adolescence has substantially advanced in recent years, the role of higher-order thinking skills in this development is, on the whole, not fully understood.

This study focuses on complex problem solving (CPS) as a prototypical skill that reflects higher-order cognitive processes. In line with Bloom’s taxonomy of higher-order thinking (Bloom et al., 1956), CPS involves analyzing, applying, synthesizing, and evaluating new information. For instance, when dealing with CPS tasks, students have to actively generate information, observe and incorporate feedback, react to changes in the problem situation that are not directly related to their own actions, adjust their own interventions, and apply self-regulation to achieve a correct solution (Funke, 2010; Wüstenberg et al., 2012). In this, CPS requires problem solvers to build complex mental representations and apply multistep solutions in opaque (i.e., intransparent) and dynamically changing problem situations and fast-paced decision-making environments (Novick and Bassok, 2005; Osman, 2010). Conceptually, CPS is composed of two overarching dimensions: knowledge acquisition and knowledge application. Knowledge acquisition describes the process of gathering information in an unknown problem situation and translating this knowledge into a mental representation of the underlying problem structure (Wüstenberg et al., 2012). Knowledge application describes the specific use of the previously gathered knowledge in an attempt to find a workable solution to the problem at hand (Novick and Bassok, 2005).

Due to the relevance of CPS and the higher-order thinking skills related to it, the arguably most acknowledged and most comprehensive international large-scale educational assessment, the Programme for International Student Assessment (PISA), included a measure of CPS in its most recent cycle in 2012 (OECD, 2014). PISA takes place in 3-years cycles and measures 15-years-old students’ achievement across more than 70 countries worldwide in mathematics, science, and reading but has increasingly focused on transversal and higher-order thinking skills. With the inclusion of CPS in PISA, the need for psychometrically sound assessment instruments became paramount, and a number of new assessment approaches to CPS were developed, among them the MicroDYN approach (Wüstenberg et al., 2012), the Genetics Lab (Sonnleitner et al., 2013), and the MultiFlux system (Kröner et al., 2005). Further, in the attempt to better understand the nomological network of CPS, cross-sectional studies have shown that CPS is related to but, at the same time, substantially different from other cognitive abilities, such as FR (Wüstenberg et al., 2012) and WM (Schweizer et al., 2013). Both FR and WM are usually considered hallmark indicators of human intellect (McGrew, 2009) and have frequently been used as developmental outcomes. Broadly, McGrew (2009, p. 5) defines FR as “the use of deliberate and controlled mental operations to solve novel problems that cannot be performed automatically,” whereas WM is understood as “the ability to apprehend and maintain awareness of a limited number of elements of information in the immediate situation.” However, there are a number of conceptual differences between CPS on the one hand and FR and WM on the other. In particular, CPS involves active and planned exploration of a dynamically changing environment going considerably beyond simple problem solving that is part of the definition of FR. These unique aspects of CPS are neither part of the definition of FR and WM nor of their operationalizations (Raven, 2000; Wüstenberg et al., 2012). In this study, FR and WM serve as predictors of CPS. Simple problem solving and inductive thinking processes that are considered part of FR are fundamental for the cognitive processes involved in CPS (Wüstenberg et al., 2012). In addition to this, WM is a conceptual precursor of CPS because it limits the amount of information that can be concurrently stored when solving a complex problem (Wirth and Klieme, 2003). However, FR is expected to be a stronger predictor of CPS because FR involves cognitive processes that are directly relevant for CPS, whereas WM only sets the upper range for the information directly accessible when no further external aid is available.

Beyond its relation to FR and WM, the relevance of CPS as an educational outcome in itself has been shown by a number of recent studies that have corroborated its empirical value in predicting outcome variables. For instance, CPS is a strong predictor of academic (Wüstenberg et al., 2012; Greiff et al., 2013b) and occupational achievement (Danner et al., 2011) and incrementally predicts these outcomes beyond FR and WM. However, despite this interest in CPS as an important outcome and its development, studies on the relations between CPS and other cognitive abilities have all been cross-sectional in nature. The only longitudinal study on CPS was conducted by Frischkorn et al. (2014). Using a sample of about 300 students, the authors showed that CPS proficiency moderately increased over a rather short period of 2 years and that FR predicted this development. However, not much more is known about the antecedents of CPS and even less about the factors that influence its development. To address this limitation, the present study aimed at providing new evidence for the development of CPS as a cognitive ability that is composed of several higher-order thinking skills (Wirth and Klieme, 2003; Griffin et al., 2012; Greiff et al., 2013b). In this, we derived two hypotheses:

Hypothesis 1 (H1): FR and WM will longitudinally predict both CPS knowledge acquisition and CPS knowledge application 3 years later.
Hypothesis 2 (H2): Compared with WM, FR will be a stronger longitudinal predictor of CPS knowledge acquisition and knowledge application.

With the aim of conceptually and empirically expanding our understanding of CPS and how it evolves, we used a large sample of Finish sixth grade students to longitudinally investigate for the first time ever how FR and WM, as two hallmark indicators of human cognition, (McGrew, 2009), influence the development of CPS in concert over a 3-year period of time. To assess CPS, we employed the MicroDYN approach that was part of the assessment of CPS in the PISA 2012 survey.

## Materials and Methods

### Participants^1^

This^1^ study initially employed a sample composed of all 2,057 sixth graders in a Southern Finnish municipality (*M* = 12.41 years, SD = 0.52; 1,051 female, 987 male, 19 missing sex) who attended school on the day of testing. Testing at Time 1 took place during weeks 17–20 in 2010. All students provided demographic data and worked on a paper-based test battery that included WM and FR measures. Testing at Time 2 was conducted during weeks 10–12 in 2013^2^. There, students worked on a computer-based online test battery that included CPS. Tests were administered by teachers either in classrooms (Time 1) or in the schools’ computer labs (Time 2).

For our analyses, we excluded the data of all students who had not worked on any of the measures used in our analyses, yielding a final data set of 2,021 students (*M =* 12.41 years, SD = 0.52; 1,041 female, 978 male, 2 missing sex). Of these 2,021 students, 1,696 participated in the panel study at Time 2 (*M =* 15.22 years, SD = 0.43; 867 female, 829 male), implying that some data were missing because families moved to another municipality, some individual students were ill on the day of testing, and the online test administration led to some technical problems, particularly on the initial days of testing (see **Table 1** for the sample size for each construct). Nevertheless, we used all available information in our analyses; that is, we included data from all 2,021 students even if not all of them provided data on each measure. Missing data varied across indicators and time with a mean of 27.2%. Little’s MCAR test revealed that data were missing completely at random (χ^2^ = 22106.051, *df* = 22333, *p* = 0.859).

**Table 1:** Means, SD, correlations, and internal consistencies of the constructs.

Construct	*M*	SD	McDonald’s ω	1	2	3	4	5	*N*
(1) Deductive reasoning (FR)^(1)^	0.49	0.23	0.64	–					1978
(2) Scientific reasoning (FR)	0.32	0.28	0.95	0.40^∗∗^	*–*				1948
(3) Working memory	0.57	0.25	0.83	0.28^∗∗^	0.32^∗∗^	*–*			1949
(4) CPS Knowledge Acquisition	0.36	0.27	0.95	0.29^∗∗^	0.32^∗∗^	0.26^∗∗^	–		1244
(5) CPS Knowledge Application	0.38	0.28	0.90	0.28^∗∗^	0.33^∗∗^	0.31^∗∗^	0.56^∗∗^	–	1106

*Means, SDs, and correlations were computed in SPSS based on manifest scores. Manifest scores were scaled to range from 0 to 1. Internal consistencies were calculated using McDonald’s (1999) ω coefficient with ω = (Σλ_*i*_)^*2*^/([Σλ_*i*_]^*2*^ + Σδ_*i*_), where λ_*i*_ are the factor loadings and δ_*ii*_ the residual variances. FR, fluid reasoning. ^(*1*)^For deductive reasoning, one item had to be excluded due to a non-significant loading on the respective factor. *^∗∗^p* < 0.001.*

### Measures

#### Working Memory

Working memory at Time 1 was measured with an adaption of the arithmetic subtest of the Wechsler Adult Intelligence Scale – Revised (WAIS-R: Wechsler, 1981). Teachers read aloud eight arithmetical problems one after another (e.g., If you buy two bus tickets and one ticket costs 3 euros 50 cents, how much money do you get back if you give 10 euros?). Students then provided written answers within predefined time limits. The items targeted children’s ability to focus and to keep information available in WM while solving arithmetical problems. According to the delineation presented by Oberauer et al. (2000), the items represented WM with a high loading on the functional factor *storage and transformation of information* within the *numerical* content category. Each item was scored as correct or incorrect (i.e., 1 or 0), resulting in eight indicators of WM.

#### Fluid Reasoning

Fluid reasoning at Time 1 was measured with one deductive and one scientific reasoning test. The first test was a subtest of the Ross Test of Higher Cognitive Processes (Ross and Ross, 1976) that targeted deductive reasoning. It has been used to assess the higher-order thinking of students in an international context (e.g., Hopson et al., 2001) and to measure cross-curricular outcomes of education in Finland (Hautamäki et al., 2010). For each of eight items, students were presented a premise (e.g., The temperature of Lake Saimaa is 5°C) and a conclusion (e.g., Lake Saimaa is too cold for swimming) and had to choose a second premise from several alternatives (e.g., Most lakes are too cold for swimming; It is wintertime; 5°C water is too cold for swimming; Lake Saimaa is always cold; Swimming in cold water is no fun) that made the conclusion true (i.e., 5°C water is too cold for swimming). The test measures deductive reasoning because it requires the ability to reason and draw specific conclusions when given general conditions (McGrew, 2009). The items were coded as correct or incorrect (i.e., 1 or 0), resulting in eight indicators of deductive reasoning. The second test was a modified version (see Hotulainen et al., 2014) of Shayer’s (1976) science reasoning task “Pendulum,” which is based on one of the formal operational schemata identified by Inhelder and Piaget (1958). The original task has been modified in a way that it includes five items that tap late concrete-level and three items that tap early formal-level thinking (Hautamäki, 1989). The context was changed from a science-type experimental situation to a well-known context of Formula 1 (see Hotulainen et al., 2014). The students’ task was to find out whether the effect of a certain variable (i.e., driver, car, tires, and track) on the time per lap could be identified. In items one to six, students were provided with a predefined set of comparisons (e.g., two different cars; same drivers, tires, and tracks) and were asked to evaluate whether the effect of the variables (e.g., cars) on the output variable could be measured accurately. For the last two tasks, the students were given a goal (e.g., to test the effect of “cars”) and were asked to mark the variables that needed to be varied to achieve the goal. This test has been used in Finnish national large-scale assessments and was validated using representative as well as small-scale samples in Finland (see Hotulainen et al., 2014). For the eight tasks that all required reflective abstraction, such as controlling and excluding certain variables, students evaluated whether the given information could produce a certain conclusion or not. The items were coded as correct or incorrect (i.e., 1 or 0), resulting in eight indicators of scientific reasoning.

#### Complex Problem Solving

Complex problem solving at Time 2 was measured with intransparent and dynamically changing problem situations that were based on the MicroDYN approach. Whereas earlier approaches measuring CPS use one complex task including a great number of problem elements, MicroDYN implements multiple tasks varying in difficulty that can be administered in less than 1 h with a small number of problem elements. MicroDYN tasks are based on linear structural equations and include usually up to three input variables that are related to up to three output variables (see description of an example item below). Advantages of the MicroDYN approach are high reliability, little dependence of performance indicators, and application of tasks with appropriate difficulty tailored to the CPS proficiency of participants (for more information on the MicroDYN approach, see Wüstenberg et al., 2012; Greiff et al., 2013b). With regard to validity, MicroDYN showed significant correlations with other CPS measures in a multitrait-multimethod approach including three different CPS tasks in which the CPS factor explained additional variance in school grades above and beyond FR (Greiff et al., 2013a). Tasks that were based on the MicroDYN approach and that were similar to tasks used in this study were applied in PISA (see OECD, 2014).

In each of the nine CPS tasks applied in this study, students were tested on their ability to generate new knowledge (i.e., CPS knowledge acquisition) and to apply this knowledge (i.e., CPS knowledge application). CPS knowledge acquisition and CPS knowledge application are considered the two core dimensions of problem solving (Novick and Bassok, 2005). Empirical research on the dimensionality of CPS has frequently shown that the two dimensions are distinct; that is, two-dimensional models result in better model fit than one-dimensional models (e.g., Kröner et al., 2005; Wüstenberg et al., 2012). CPS knowledge acquisition and not CPS knowledge application measured with MicroDYN was also shown to explain variance in grade point average, even beyond reasoning measures (Greiff et al., 2014). These results emphasize the importance of considering both dimensions instead of a second-order CPS factor that is composed of CPS knowledge acquisition and CPS knowledge application.

**FIGURE 1:**
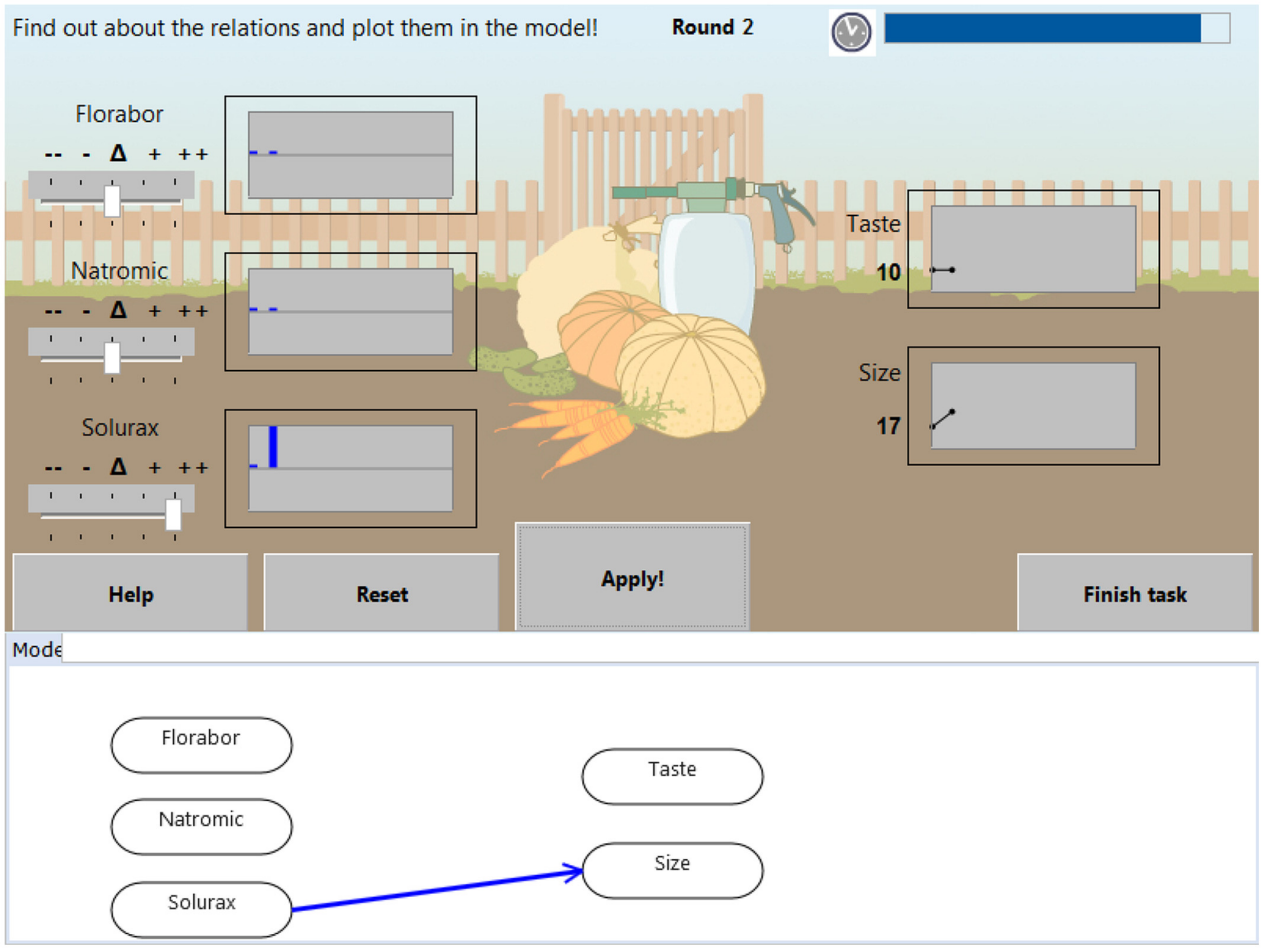
**Screenshot of the MicroDYN item *PlantingPumpkins* in the knowledge acquisition phase.** Students are asked to manipulate the input variables Florabor, Natromic, and Solurax (see left upper side) to find out how they are related to the two output variables Taste and Size (see right upper side). Simultaneously to the exploration, students are asked to draw relations between variables in the mental model as they suppose.

In MicroDYN, each of the nine CPS tasks consisted of one knowledge acquisition item and one knowledge application item, which were administered separately in two distinct phases. For instance, in the task *Planting Pumpkins* (see **Figure 1**), the knowledge acquisition item asked students to find out how input variables (e.g., fertilizers with fictitious names such as Florabor, Natromix, Solurax) were related to output variables (e.g., the size and taste of the pumpkins; **Figure 1**). The students identified the relations by moving sliders that changed the amounts of the input variables (e.g., increasing the use of the fertilizer Solurax) and by observing the effect on the output variables. However, the state of the output variables could also change independent of students’ actions (e.g., the size of a pumpkin could increase by itself). While engaging in this active exploration, the students also had to draw a causal model to represent the assumed relations between the variables. CPS knowledge acquisition was scored as correct or incorrect (i.e., 1 or 0) depending on the accuracy of the students’ model. In the knowledge application phase, the correct model was presented to students, and they had to achieve given target goals in a maximum of four steps. For instance, students had to increase the size of the pumpkins. CPS knowledge application was scored as correct or incorrect (i.e., 1 or 0) depending on whether all goals were reached. In total, CPS was measured with 18 items (i.e., nine for CPS knowledge acquisition and nine for CPS knowledge application).

#### Gender

Gender was used as a covariate in all models with female students being categorized as 1 and male students as 2.

### Statistical Analyses

We used structural equation modeling in Mplus 7.1 (Muthén and Muthén, 2010) and SPSS for descriptive statistics. Weighted least squares mean and variance adjusted (WLSMV) estimation with pairwise present approach was used for parameter estimation in structural equation models because the manifest indicators of WM, FR, and CPS were dichotomous (Muthén and Muthén, 2010)^3^. Measurement models of constructs were evaluated using confirmatory factor analysis. Residuals of manifest indicators were modeled as not being correlated in all analyses. Model fit was evaluated by applying standard fit indices such as the confirmatory fit index (CFI; values greater than 0.95 indicate good fit; values greater than 0.90 indicate acceptable fit; Hu and Bentler, 1999) and the root mean square error of approximation (RMSEA; values less than 0.06 indicate good fit; values less than 0.08 indicate acceptable fit; Marsh et al., 2005).

**FIGURE 2:**
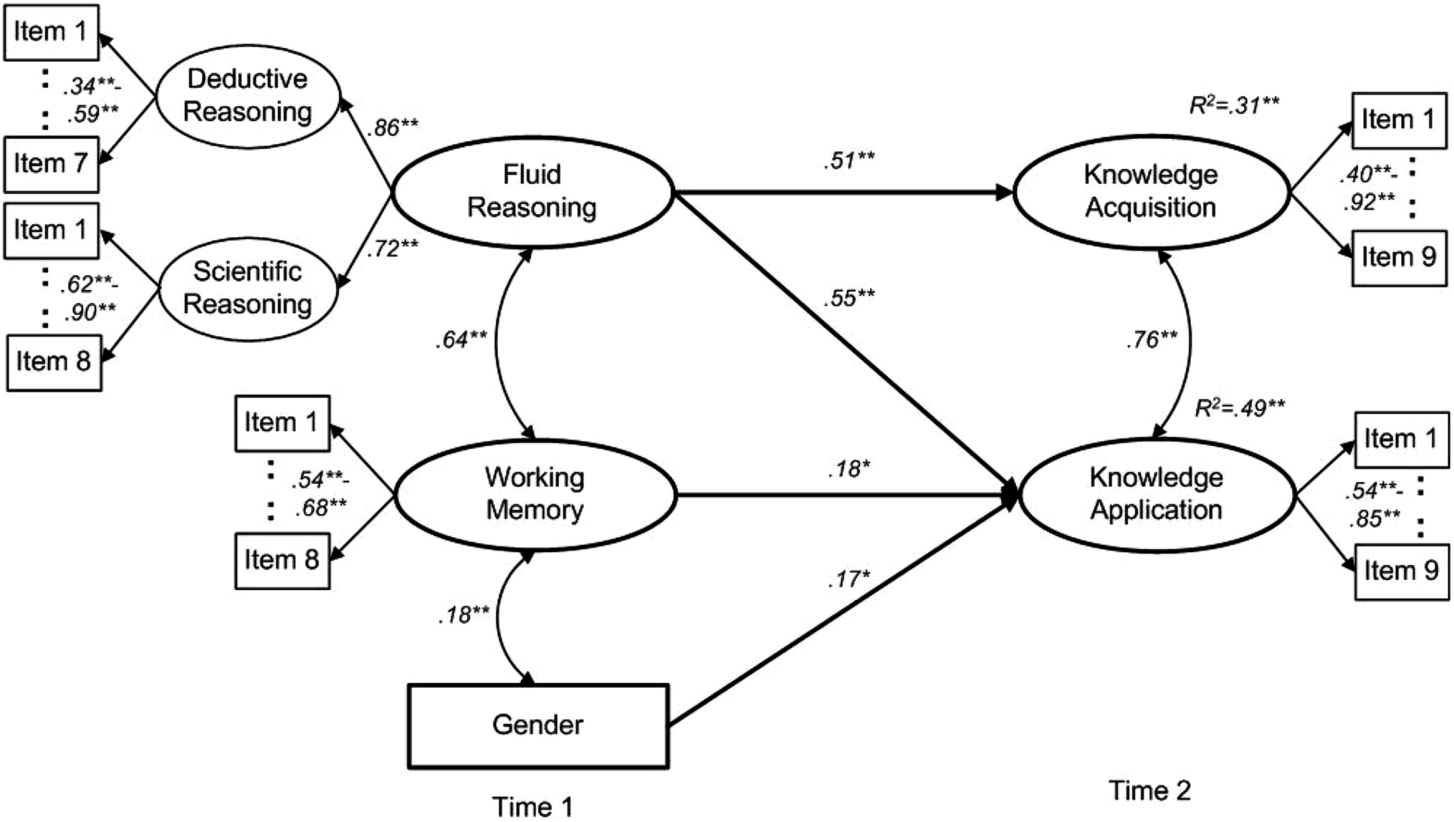
**The longitudinal prediction of CPS knowledge acquisition and CPS knowledge application by fluid reasoning (FR) and working memory (WM; Model A).** We controlled for gender by regressing CPS knowledge acquisition and CPS knowledge application on gender; gender was allowed to correlate with FR and WM; only significant paths are displayed (i.e., effect sizes of non-significant paths are mentioned in the text). Gender was not significantly correlated with FR (*r* = -0.058, SE = 0.03, 95% CI [-0.115, 0.000], *p* = 0.05). Ellipses represent latent constructs; rectangles represent measured variables. Numbers adjacent to paths are standardized coefficients. Numbers adjacent to the item indicators of latent constructs represent the range of factor loadings. WLSMV estimation was used for parameter estimation. Gender categories are: 1 = female; 2 = male. ^∗^*p* < 0.05, ^∗∗^*p* < 0.001.

We used the baseline Model A as starting point for testing our two hypotheses (see **Figure 2**), in which we utilized WM (measured with eight items) and a second-order FR factor including scientific reasoning (eight items) and deductive reasoning (eight items) as predictors. FR and WM were allowed to correlate. Gender (as a covariate) was used as an additional predictor of CPS knowledge acquisition and CPS knowledge application and was allowed to correlate with FR and WM. CPS knowledge acquisition (nine items) and CPS knowledge application (nine items) were used as criteria and were allowed to correlate.

To investigate Hypothesis 1, Model A was tested against two alternative models in which either the effect of WM on both CPS dimensions was constrained to be zero (i.e., Model B) or the effect of FR on both CPS dimensions was constrained to be zero (i.e., Model C). These constraints resulted in more parsimonious models that would be preferred if the fit of the respective model was not significantly worse than the fit of Model A, implying that one of the predictors may not be needed in the longitudinal prediction of CPS. To investigate Hypothesis 2, Model A was tested against Model D, in which the effects of WM and FR were constrained to equality. If Model A did not show a significantly better fit than Model D, then FR could not be considered stronger than WM in predicting CPS.

## Results

Descriptive statistics and manifest correlations are presented in **Table 1**, revealing significant relations between all the constructs that were employed to test the longitudinal relations. To measure internal consistency, McDonald’s ω was applied (**Table 1**). McDonald’s ω takes size of factor loadings into account when estimating the proportion of test variance due to the latent factors (Zinbarg et al., 2005). Internal consistencies were appropriate for population-level analyses.

With regard to CPS, a two-dimensional model including CPS knowledge acquisition and CPS knowledge application as separate factors (χ^2^ = 307.347, *df* = 134, *p* < 0.001; CFI = 0.988, RMSEA = 0.032) revealed a better fit (χ^2^-difference test^4^ = 84.965, *df* = 1, *p* < 0.001) than a one-dimensional model (χ^2^ = 446.524, *df* = 135, *p* < 0.001; CFI = 0.978, RMSEA = 0.043), in which all items were combined under one first-order factor. In this two-dimensional measurement model, CPS knowledge acquisition and CPS knowledge application were highly correlated (*r* = 0.84, *p* < 0.001). The one-dimensional measurement model of WM revealed good fit (χ^2^ = 38.209, *df* = 20, *p* = 0.008; CFI = 0.990, RMSEA = 0.022), whereas the second order factor model for FR with scientific reasoning and deductive reasoning as first order factors revealed acceptable fit with regard to CFI but not with regard to RMSEA (χ^2^ = 1535.683, *df* = 89, *p* < 0.001; CFI = 0.911, RMSEA = 0.090). In the measurement model of deductive reasoning one item had to be excluded due to a non-significant factor loading (β = 0.002, *p* = 0.97). However, this measurement model for FI fit significantly better (χ^2^-difference test = 129.114, *df* = 1, *p* < 0.001) than a one-dimensional model (χ^2^ = 1729.559, *df* = 90, *p* < 0.001; CFI = 0.899, RMSEA = 0.095). In summary, we used a two-dimensional model for CPS, a second order model for FI, and a one-dimensional model for WM when conducting analyses with several constructs in structural equation modeling.

With Hypothesis 1, we expected that both FR and WM would longitudinally predict both CPS knowledge acquisition and CPS knowledge application 3 years later while controlling for gender differences (see Model A depicted in **Figure 2**). The model showed a good overall fit with regard to RMSEA and adequate fit with regard to CFI (see **Table 2**). The predictors WM and FR were strongly related to each other, *r* = 0.638, SE = 0.03, 95% CI [0.576, 0.700], *p* < 0.001. Crucially for longitudinal development, CPS knowledge acquisition was predicted by FR (β = 0.514, SE = 0.07, 95% CI [0.38, 0.65], *p* < 0.001), but not by WM (β = 0.067, SE = 0.07, 95% CI [-0.07, 0.20], *p* = 0.34), and CPS knowledge application was predicted by both FR (β = 0.550, SE = 0.08, 95% CI [0.40, 0.70], *p* < 0.001) and WM (β = 0.180, SE = 0.08, 95% CI [0.03, 0.33], *p* = 0.02) while controlling for gender. Gender, which was considered as covariate in our analyses, was related to WM (β = 0.177, SE = 0.03, *p* < 0.001) and CPS knowledge application (β = 0.174, SE = 0.04, *p* < 0.001) indicating a somewhat higher performance for male students, but not to FR (β = -0.058, SE = 0.03, *p* = 0.05) and CPS knowledge acquisition (β = 0.061, SE = 0.03, *p* = 0.07). Further, Model A showed a better fit than both alternative Models B and C (see the χ^2^-difference tests in **Table 2**). That is, the models in which either the significant effect of WM on CPS knowledge application was constrained to zero (Model B) or the significant effects of FR on both CPS dimensions were constrained to zero (Model C) showed a worse fit than the model that included both predictors (Model A). These results clearly point toward the importance of WM and FR for the development of CPS. In summary, Hypothesis 1 was supported except that WM predicted only CPS knowledge application.

**Table 2:** Goodness of fit indices for structural models.

Structural models	*χ^2^*	*df*	*p*	CFI	RMSEA	Compare with	Δχ2^(1)^	Δ*df*^(1)^	*p*
Model A: Final model (see **Figure 2**)	2402.311	808	<0.001	0.947	0.031	*–*	*–*	*–*	*–*
Model B: WM path to CPS knowledge application constrained to zero	2409.365	809	<0.001	0.947	0.031	Model A	12.924	1	< 0.001
Model C: FR paths to CPS outcomes constrained to zero	2430.892	810	<0.001	0.946	0.031	Model A	45.214	2	< 0.001
Model D: FR and WM paths to CPS outcomes constrained to be equal	2439.703	810	<0.001	0.946	0.032	Model A	21.717	2	< 0.001

*Models B–D represents alternative models that were identical to Model A (see **Figure 2**) except that specific paths were constrained. The χ^*2*^ and *df* values were estimated with the WLSMV estimator to obtain the CFI and RMSEA as fit indices. ^(*1*)^Differences in model fit were estimated using the χ^*2*^-difference test procedure in Mplus (see Muthén and Muthén, 2010) because χ^*2*^-differences between models could not be compared directly by subtracting χ^*2*^s and *df*s when the WLSMV estimator was used. WM, working memory; FR, fluid reasoning; CPS, complex problem solving.*

With Hypothesis 2, we expected that FR would predict the two CPS dimensions more strongly than WM would while controlling for gender. First, path coefficients of FR predicting CPS dimensions were higher than the path coefficients of WM predicting CPS dimensions in Model A. This pointed toward a stronger effect of FR. To test whether this difference was significant, we compared the model fit of Model A with the fit of the alternative Model D, in which the effects of WM and FR were constrained to equality (see **Table 2**). Again, Model A revealed a significantly better fit than the alternative Model D.

In summary, Hypothesis 1 was (mostly) supported and Hypothesis 2 was supported, showing that FR longitudinally predicted CPS knowledge acquisition, both FR and WM longitudinally predicted CPS knowledge application, and FR was a stronger predictor of CPS than WM. These findings provide support for longitudinal links between these two cognitive abilities and CPS.

## Discussion

This study set out to investigate the longitudinal relations between FR and WM measured at Time 1 as predictors of CPS knowledge acquisition and CPS knowledge application measured 3 years later at Time 2. The results of our test of Hypothesis 1 revealed that FR was a precursor of both CPS dimensions in a model that controlled for gender as covariate, whereas WM was related only to CPS knowledge application and not to CPS knowledge acquisition when considered together with FR. Further, as indicated by the results of our test of Hypothesis 2, FR was a stronger predictor of CPS than WM was, thus highlighting the particular importance of FR for the longitudinal development of both CPS knowledge acquisition and CPS knowledge application. Gender as a covariate showed small positive relations to WM and CPS knowledge application, indicating that boys somewhat outperformed girls.

This pattern of results provides further support for the well-known finding that fundamental cognitive abilities serve as precursors of advanced cognitive abilities later in life (e.g., Rose et al., 2008; Bornstein, 2014) and that cognitive development increasingly involves higher-order thinking skills as children grow older (Galotti, 2011; Albert and Steinberg, 2011). At the same time, the current study extends the existing literature on the development of human cognition by introducing CPS as developmental outcome that involves aspects of complex cognition (Funke, 2010) and that seems to evolve on the basis of fundamental cognitive abilities, such as FR and WM, as shown in our test of Hypothesis 1. These findings also have implications for educational policy and teaching. As already noted, making students good problem solvers is key to good education (Mayer and Wittrock, 2006) and CPS as a transversal skill has recently received attention in large-scale assessments such as PISA (OECD, 2014). It is therefore of utmost importance to increase understanding of how CPS skills emerge and manifest themselves. Undeniably, the identification of developmental trajectories is a necessary prerequisite for any attempt to improve children’s higher-order thinking skills.

In addition to enriching our understanding of the development of the human mind, the current study complements existing research on CPS that has primarily been based on cross-sectional studies. For instance, cross-sectional studies provided initial evidence that CPS is related to both FR and WM, although more strongly to FR (cf. Wüstenberg et al., 2012; Schweizer et al., 2013). The pattern of results reported here provides further support for the strong relevance of FR and the somewhat less relevance of WM when longitudinally predicting CPS as shown in our test of Hypothesis 2. Interestingly, in this study, WM was related only to CPS knowledge application but not to CPS knowledge acquisition. This might be due to the specific operationalization of MicroDYN as the measure of CPS. During the assessment of CPS knowledge acquisition, students were allowed to draw a causal model in MicroDYN while they explored the problem space, reducing the load on WM. In the assessment of CPS knowledge application, students had to coordinate their manipulations of the variables to reach certain goals, requiring them to mentally consider the effect of multiple interventions simultaneously and to think several steps ahead. Thereby, a higher cognitive load was produced in the CPS knowledge application phase than in the CPS knowledge acquisition phase. This might explain why only knowledge application was significantly predicted by WM even though FR was a notably stronger predictor of CPS (Hypothesis 2).

There are several limitations to this study that need to be considered. For instance, the WM assessment we applied contained WM tasks with arithmetical content. In turn, this content might be related to math proficiency and math anxiety and would hence not offer a pure measure of WM. To this end, we suggest that future studies include broader and more diverse measures of WM that are not associated with arithmetic skills. A second limitation of the current research is that we did not control for initial CPS level at Time 1. However, investigating cross-lagged relations between constructs is mandatory for testing more advanced theoretical models such as the cognitive cascade model (Fry and Hale, 1996). The cognitive cascade model assumes that human cognition advances from fundamental cognitive abilities in infancy to a complex pattern of higher-order thinking skills in adolescence in an increasingly complex sequence of steps in which previous abilities lay the foundation for abilities that develop later. It falls to future studies to test these types of more complex theoretical suppositions in the attempt to paint a detailed picture of how CPS evolves. Future studies could additionally address important questions about critical periods for intervention and developmental differences between boys and girls in CPS proficiency and CPS development.

Even though the current results offer only a starting point, they provide strong evidence for an evolving chain in which developmental change in fundamental cognitive abilities in childhood undergirds higher-order thinking in adolescence. This result clearly supports the view that cognitive development evolves from basic processes in childhood into an increasingly complex structure of higher-order thinking skills in adolescence that enable the human mind to perform highly complex cognitive operations.

## Conflict of Interest Statement

The authors declare that the research was conducted in the absence of any commercial or financial relationships that could be construed as a potential conflict of interest.

## Footnotes

1 This study was part of an official municipal evaluation of learning to learn. The cohort study was commissioned and the research design with scales were approved by the local Education Department. The same scales and design have been used also in national educational evaluations commissioned by the Ministry of Education and Culture, and by the Finnish National Board of Education, based on the Basic Education Act (1999). The measures and design have been approved, in relation with another study, also by the Ethical Committee of Finnish National Institute for Health and Welfare. In the beginning of the longitudinal follow-up study, all parents were informed by a letter.

2 The data were drawn from a panel study. The sample gathered at Time 2 was used in other publications (e.g., Vainikainen, 2014; Wüstenberg et al., 2014), for instance, to investigate cross-sectional relations between CPS and other constructs. This paper includes the first longitudinal analyses that combined the data collection (including CPS) in 2013 with earlier data collection. Thus, the analyses that longitudinally evaluate the determinants of CPS knowledge acquisition and CPS knowledge application are unique.

3 We also ran an additional model in which we used imputed data (i.e., TYPE = basic in Mplus with five imputed datasets). This additional model yielded comparable results that did not differ substantially from analyses based on data without imputations. However, difftest procedures for model comparisons in Mplus are not available for imputed data. Thus, we present results based on non-imputed data in this paper to be able to compare Models A through D.

4 Differences in model fit were estimated using the χ^2^-difference test procedure in Mplus (see Muthén and Muthén, 2010) because χ^2^-differences between models could not be compared directly by subtracting χ^2^s and *df*s when the WLSMV estimator was used.

## References

[B1] Albert D. Steinberg L. (2011). Age differences in strategic planning as indexed by the Tower of London. *Child Dev.* 82 1501–1517. 10.1111/j.1467-8624.2011.01613.x 21679178

[B2] Asato M. R. Sweeney J. A. Luna B. (2006). Cognitive processes in the development of TOL performance. *Neuropsychologia* 44 2259–2269. 10.1016/j.neuropsychologia.2006.05.010 16797612

[B3] Autor D. H. Levy F. Murnane R. J. (2003). The skill content of recent technological change: an empirical exploration. *Q. J. Econ.* 118 1279–1333. 10.1162/003355303322552801

[B4] Blaga O. M. Shaddy D. J. Anderson C. J. Kannass K. N. Little T. D. Colombo J. (2009). Structure and continuity of intellectual development in early childhood. *Intelligence* 37 106–113. 10.1016/j.intell.2008.09.003 20046219 PMC2631272

[B5] Bloom B. S. Engelhart M. D. Furst E. J. Hill W. H. Krathwohl D. R. (1956). *Taxonomy of Educational Objectives: The Classification of Educational Goals. The Cognitive Domain*. New York, NY: David McKay.

[B6] Bornstein M. H. (2014). Infancy and the rest of the lifespan. *Annu. Rev. Psychol.* 65 121–158. 10.1146/annurev-psych-120710-100359 24405360 PMC5865600

[B7] Bornstein M. H. Hahn C.-S. Bell C. Haynes O. M. Slater A. Golding J. (2006). Stability in cognition across early childhood. *Psychol. Sci.* 17 151–158. 10.1111/j.1467-9280.2006.01678.x 16466423

[B8] Bornstein M. H. Hahn C.-S. Wolke D. (2013). Systems and cascades in cognitive development and academic achievement. *Child Dev.* 84 154–162. 10.1111/j.1467-8624.2012.01849.x 22974268 PMC3525805

[B9] Conklin H. M. Luciana M. Hooper C. J. Yarger R. S. (2007). Working memory performance in typically developing children and adolescents. Behavioral evidence of protracted front lobe development. *Dev. Neuropsychol.* 31 103–128. 10.1207/s15326942dn3101_6 17305440

[B10] Danner D. Hagemann D. Schankin A. Hager M. Funke J. (2011). Beyond IQ. A latent state trait analysis of general intelligence, dynamic decision making, and implicit learning. *Intelligence* 39 323–334. 10.1016/j.intell.2011.06.004

[B11] Demetriou A. Mouyi A. Spanoudis G. (2008). Modeling the structure and development of g. *Intelligence* 36 437–454. 10.1016/j.intell.2007.10.002

[B12] Frischkorn G. Greiff S. Wüstenberg S. (2014). The development of complex problem solving: a latent growth curve analysis. *J. Edu. Psychol.* 106 1004–1020. 10.1037/a0037114

[B13] Funke J. (2010). Complex problem solving: a case for complex cognition? *Cogn. Process*. 11 133–142. 10.1007/s10339-009-0345-0 19902283

[B14] Fry A. F. Hale S. (1996). Processing speed, working memory, and fluid intelligence. *Psychol. Sci.* 7 237–241. 10.1111/j.1467-9280.1996.tb00366.x 11035218

[B15] Galotti K. M. (2011). *Cognitive Development*. Thousand Oaks, CA: SAGE.

[B16] Greiff S. Fischer A. Wüstenberg S. Sonnleitner P. Brunner M. Martin R. (2013a). A multitrait-multimethod study of assessment instruments for Complex Problem Solving. *Intelligence* 41 579–596. 10.1016/j.intell.2013.07.012

[B17] Greiff S. Wüstenberg S. Molnar G. Fischer A. Funke J. Csapo B. (2013b). Complex problem solving in educational settings – something beyond g: concept, assessment, measurement invariance, and construct validity. *J. Edu. Psychol.* 105 364–379. 10.1037/a0031856

[B18] Greiff S. Kretzschmar A. Müller J. C. Spinath B. Martin R. (2014). The computer-based assessment of complex problem solving and how it is influenced by students’ information and communication technology literacy. *J. Educ. Psychol.* 106 666–680. 10.1037/a0035426

[B19] Griffin P. McGaw B. Care E. (2012). *Assessment and Teaching of 21st Century Skills*. Dordrecht: Springer 10.1007/978-94-007-2324-5

[B20] Hautamäki J. (1989). “The application of a Rasch model on Piagetian measures of stages of thinking,” in *Adolescent Development and School Science* eds Adey P. Bliss J. Head J. Shayer M. (London: Falmers).

[B21] Hautamäki A. Hautamäki J. Kupiainen S. (2010). “Assessment in schools – learning to learn,” in *International Encyclopedia of Education* Vol. 3 eds Peterson P. Baker E. McGaw B. (Oxford: Elsevier) 268–272. 10.1016/B978-0-08-044894-7.00323-7

[B22] Hoff E. Tian C. (2005). Socioeconomic status and cultural influences on language. *J. Commun. Disord.* 38 271–278. 10.1016/j.jcomdis.2005.02.003 15862810

[B23] Hopson M. H. Simms R. L. Knezek G. A. (2001). Using technology-enriched environments to improve higher-order thinking skills. *J. Res. Technol. Edu.* 34 109–120. 10.1080/15391523.2001.10782338

[B24] Hotulainen R. Thuneberg H. Hautamäki J. Vainikainen M.-P. (2014). Measured attention in prolonged over-learned response tasks and its correlation to high level scientific reasoning and school achievement. *Psychol. Test Assess. Model.* 56 237–254.

[B25] Hu L. Bentler P. M. (1999). Cutoff criteria for fit indexes in covariance structure analysis: conventional criteria versus new alternatives. *Struct. Equ. Model.* 6 1–55. 10.1080/10705519909540118

[B26] Inhelder B. Piaget J. (1958). *The Early Growth of Logic in the Child*. London: Routledge & Kegan Paul.

[B27] Kavsek M. (2004). Predicting later IQ from infant visual habituation and dishabituation: a meta-analysis. *J. Appl. Dev. Psychol.* 25 369–393. 10.1016/j.appdev.2004.04.006

[B28] Kröner S. Plass J. L. Leutner D. (2005). Intelligence assessment with computer simulations. *Intelligence* 33 347–368. 10.1016/j.intell.2005.03.002

[B29] Marsh H. Hau K. Grayson D. (2005). “Goodness of fit evaluation in structural equation modeling,” in *Contemporary Psychometrics* eds Maydeu-Olivares A. McArdle J. (Mahwah, NJ: Erlbaum) 275–340.

[B30] Mayer R. E. Wittrock M. C. (2006). “Problem solving,” in *Handbook of Educational Psychology* eds Alexander P. A. Winne P. H. (Mahwah, NJ: Lawrence Erlbaum) 287–303.

[B31] McDonald R. P. (1999). *Test Theory: A Unified treatment*. Mahwah, NJ: Erlbaum.

[B32] McGrew K. S. (2009). CHC theory and the human cognitive abilities project. Standing on the shoulders of the giants of psychometric intelligence research. *Intelligence* 37 1–10. 10.1016/j.intell.2008.08.004

[B33] Muthén L. K. Muthén B. O. (2010). *Mplus User’s Guide* 6th Edn. Los Angeles, CA: Author.

[B34] Novick L. R. Bassok M. (2005). “Problem solving,” in *The Cambridge Handbook of Thinking and Reasoning* eds Holyoak K. J. Morrison R. G. (Cambridge, NY: University Press) 321–349.

[B35] Oberauer K. Süß H.-M. Schulze R. Wilhelm O. Wittmann W. W. (2000). Working memory capacity. Facets of a cognitive ability construct. *Personal. Ind. Diff.* 29 1017–1045. 10.1016/S0191-8869(99)00251-2

[B36] OECD. (2012). *Better Skills, Better Jobs, Better Lives. A Strategic Approach to Skills Policies*. Paris: OECD Publishing.

[B37] OECD. (2014). *PISA 2012 Results: Creative Problem Solving*. Paris: OECD Publishing.

[B38] Osman M. (2010). Controlling uncertainty: a review of human behavior in complex dynamic environments. *Psychol. Bull.* 136 65–86. 10.1037/a0017815 20063926

[B39] Raven J. (2000). Psychometrics, cognitive ability, and occupational performance. *Rev. Psychol.* 7 51–74.

[B40] Rose S. A. Feldman J. F. Jankowski J. J. Van Rossem R. (2008). A cognitive cascade in infancy. Pathways from prematurity to later mental development. *Intelligence* 36 367–378. 10.1016/j.intell.2007.07.003 19122757 PMC2504323

[B41] Ross J. D. Ross C. M. (1976). *Ross Test of Higher Cognitive Processes*. Novato, CA: Academic Therapy Publications.

[B42] Schweizer F. Wüstenberg S. Greiff S. (2013). Do complex problem solving dimensions measure something over and above working memory capacity? *Lear. Ind. Diff*. 24 42–52. 10.1016/j.lindif.2012.12.011

[B43] Shayer M. (1976). The pendulum problem. *Br. J. Edu. Psychol.* 46 85–87. 10.1111/j.2044-8279.1976.tb02989.x

[B44] Sonnleitner P. Keller U. Martin R. Brunner M. (2013). Students’ complex problem-solving abilities: their structure and relations to reasoning ability and educational success. *Intelligence* 41 289–305. 10.1016/j.intell.2013.05.002

[B45] Tong S. Baghurst P. Vimpani G. McMichael A. (2007). Socioeconomic position, maternal IQ, home environment, and cognitive development. *J. Pediatr.* 151 284–288. 10.1016/j.jpeds.2007.03.020 17719939

[B46] Vainikainen M.-P. (2014). *Finnish Primary School Pupils*’ *Performance in Learning to Learn Assessments: A Longitudinal Perspective on Educational Equity*. University of Helsinki, Department of Teacher Education Research Report 360. Helsinki: Unigrafia.

[B47] Wechsler D. (1981). *WAIS-R: Manual: Wechsler Adult Intelligence Scale – Revised*. Cleveland, OH: Harcourt Brace Jovanovich for Psychological Corp.

[B48] Wirth J. Klieme E. (2003). Computer-based assessment of problem solving competence. *Assess. Edu. Princ. Policy Pract.* 10 329–345. 10.1080/0969594032000148172

[B49] Wüstenberg S. Greiff S. Funke J. (2012). Complex Problem Solving. More than reasoning? *Intelligence* 40 1–14. 10.1016/j.intell.2011.11.003

[B50] Wüstenberg S. Stadler M. Hautamäki J. Greiff S. (2014). The role of strategy knowledge for the application of strategies in complex problem solving tasks. *Technol. Knowl. Learn*. 19 127–146. 10.1007/s10758-014-9222-8

[B51] Zinbarg R. E. Revelle W. Yovel I. Li W. (2005). Cronbach’s α, Revelle’s β, and McDonald’s ω. Their relations with each other and two alternative conceptualizations of reliability. *Psychometrika* 70 123–133. 10.1007/s11336-003-0974-7

